# A systems approach to identifying correlated gene targets for the loss of colour pigmentation in plants

**DOI:** 10.1186/1471-2105-12-343

**Published:** 2011-08-17

**Authors:** Sangaalofa T Clark, Wynand S Verwoerd

**Affiliations:** 1 Centre for Advanced Computational Solutions (CfACS), Agriculture and Life Sciences Division Lincoln University, Canterbury, New Zealand

## Abstract

**Background:**

The numerous diverse metabolic pathways by which plant compounds can be produced make it difficult to predict how colour pigmentation is lost for different tissues and plants. This study employs mathematical and *in silico *methods to identify correlated gene targets for the loss of colour pigmentation in plants from a whole cell perspective based on the full metabolic network of *Arabidopsis*. This involves extracting a self-contained flavonoid subnetwork from the AraCyc database and calculating feasible metabolic routes or elementary modes (EMs) for it. Those EMs leading to anthocyanin compounds are taken to constitute the anthocyanin biosynthetic pathway (ABP) and their interplay with the rest of the EMs is used to study the minimal cut sets (MCSs), which are different combinations of reactions to block for eliminating colour pigmentation. By relating the reactions to their corresponding genes, the MCSs are used to explore the phenotypic roles of the ABP genes, their relevance to the ABP and the impact their eliminations would have on other processes in the cell.

**Results:**

Simulation and prediction results of the effect of different MCSs for eliminating colour pigmentation correspond with existing experimental observations. Two examples are: i) two MCSs which require the simultaneous suppression of genes DFR and ANS to eliminate colour pigmentation, correspond to observational results of the same genes being co-regulated for eliminating floral pigmentation in *Aquilegia *and; ii) the impact of another MCS requiring CHS suppression, corresponds to findings where the suppression of the early gene CHS eliminated nearly all flavonoids but did not affect the production of volatile benzenoids responsible for floral scent.

**Conclusions:**

From the various MCSs identified for eliminating colour pigmentation, several correlate to existing experimental observations, indicating that different MCSs are suitable for different plants, different cells, and different conditions and could also be related to regulatory genes. Being able to correlate the predictions with experimental results gives credence to the use of these mathematical and *in silico *analyses methods in the design of experiments. The methods could be used to prioritize target enzymes for different objectives to achieve desired outcomes, especially for less understood pathways.

## Background

Flavonoids [1,2] are ubiquitous secondary plant metabolites that play a variety of roles in the reproduction and protection of plants. Their most obvious and perhaps best characterized role involves anthocyanins, which is a major subgroup that assists plants in attracting pollinators and seed dispersers by providing red to blue pigmentation in plant tissues. Flavonoids are among the most studied and best understood compounds in plant science with a vast amount of literature available for them. A review of the history of their research can be seen in [3,4].

Because of their role in pigmentation, changes in the expression of anthocyanins are easily observed. One example is where flowers have been found to lose their colour in response to different insect pollinators [5]. It is reasonable to expect that such loss-of-function adaptations are relatively unconstrained because they can be achieved in many ways. For example, the suppression of any one of the enzymes catalyzing a series of reactions that make up a pathway will remove its final product. So it comes as a surprise that an experimental study by Whittal et al [5] found that for independent events causing loss of colour in different populations of *Aquilegia*, the particular subset of genes that were suppressed, was strongly correlated. They also observed that the genes concerned (DFR and ANS) were co-regulated, one of similar findings of co-regulation in the anthocyanin biosynthetic pathway (ABP) genes, another example of which is made by Durbin et al. [6] who showed that colour phenotypes in plants were closely associated with changes in the regulation of gene expression.

This study complements the biological investigations from a mathematical and *in silico *perspective. Rather than observing genes and inferring network properties, we start from a known self-contained metabolic network containing all reactions necessary for the synthesis of flavonoid compounds. As reactions are catalyzed by enzymes which are coded by genes, the reactions can be correlated to their corresponding genes; without needing knowledge about the genes involved, metabolic pathway analysis (MPA) [7,8] tools are used to investigate the role of flavonoid genes in plant colour pigmentation by identifying the reactions involved and relating these to their corresponding genes. The investigation is done, first, in parallel to the biological investigations mentioned above and then expanding into other studies that provide further information.

In their work, Whittal et al remarked that the DFR and ANS enzymes corresponded to steps that were late in the ABP. This led them to interpret their finding as a consequence of pleiotropic functions for enzymes such as CHS and CHI that occur earlier in the ABP. While this explanation seems plausible enough in terms of a linear pathway, it needs to be recognized that, like all empirical pathways, the ABP is in fact embedded in a complex biochemical network. This raises additional questions, for example, even if the early enzymes perform other functions, would their loss destroy those functions or are there alternative paths available? Instead of blocking a "late" gene, would it not be more efficient to block an "early" gene and eliminate the subsequent reactions? Is the number of reactions in which an enzyme participate an adequate measure of whether it is essential, or can more refined measures be employed?

In order to answer such questions, a more quantitative approach based on the biochemical network analysis methods is clearly needed. The MPA methods are based on elementary modes (EMs) which are non-decomposable pathways or routes that lead from an external substrate to an external product with the network maintained at steady state. The EMs consist of minimal sets of reactions that allow the system to operate at steady state [9,10]; they are based on the structure of the network and thus defined in the context of whole-cell metabolism.

EMs can be used to compute minimal cut sets (MCSs) [11]. MCSs are the minimal sets of reactions that, when blocked, will repress a certain metabolic functionality; the functionality can be characterized by a set of EMs, which is then used to calculate the MCSs used here to study structural fragility and identify knock out strategies for plant pigmentation.

By performing a comprehensive mathematical analysis of the extracted subnetwork structure, the study enables the prediction of all possible EMs and MCSs compatible with the network structure. Such exhaustive characterization is very hard to achieve experimentally because, regardless of how many examples of a phenomenon one has observed, there might always be others not yet observed. However, the mathematical analysis provides a complete set of all possible EMs and MCSs. It is this aspect of completeness, subject only to a complete knowledge of the network itself, that makes it possible to make quantitative assessments e.g. of the relative importance of reactions and their corresponding enzymes/genes.

Hence, the study provides an interesting look at all the possible target genes for anthocyanin suppression and how they fit into different situations. For example, although it is possible to target one single enzyme in order to suppress anthocyanin production, the question is whether that would be best? In nature, the absence of more than one enzyme is often observed and the reasons for pigmentation loss are numerous: it can be a single mutation in CHS, as described for a specific white flowering Matthiola line [12] or petunia [13], or a mutation in a regulatory gene affecting a single flavonoid gene[14], or many flavonoid genes[15]. In the same plant species only one enzyme might be affected or many enzymes can be affected [16-18]. This multiplicity of possible strategies and exploring the related implications, using MPA methods such as MCSs, is a focus of this study.

The study proceeds through a number of stages: First, a self-contained flavonoid subnetwork is extracted from the full *Arabidopsis *network. Next, the set of EMs for it are calculated. For the main focus of the study, which is the role of anthocyanin genes in the loss of colour pigmentation, the EMs that directly lead to the formation of anthocyanin compounds are identified and taken to constitute the ABP. MCSs and other methods designed for EM-based network structural analysis are then applied to identify and compare candidate genes (through their associated reactions) that could be targeted for eliminating colour pigmentation. Finally, we also use flux balance analysis (FBA) to investigate how each MCS candidate would affect the rate of production of associated metabolites. Where possible, the analysis results are compared and related to results from biological studies.

## Methods

### Extracting the subnetwork

The standard model plant, *Arabidopsis*, is used for the study because of its fully sequenced genome and the actively curated metabolic data (AraCyc) [19] available for it. The source data is the full genome-scale *Arabidopsis *network formed by collecting all known metabolic reactions and enzymes from the AraCyc database version 8.0 [20]. A set of custom designed AWK [21] scripts [22] performs the parsing of AraCyc into an SBML [23] network specification, integrating reaction direction information dispersed in reaction, enzyme and pathway tables of the database. This network specification file is used as input for the CellNetAnalyzer (CNA) program [24], a software application that offers the capacity to conduct structural and functional analysis of the network (the 4 additional files: 1) *AC8_subnet.sbml*, 2) *metabolites*, 3) *prelim_constr_influx.val*, and 4) *reactions*, constitute input files for CNA).

From the MPA perspective, a metabolic network is a (hyper-) graph where the nodes represent compounds and the edges chemical reactions. The boundary nodes are external biochemical inputs (substrates) or outputs (products) while the internal nodes are intermediate compounds with associated stoichiometric constraints to guarantee mass conservation. Full specification of the network also requires identification of the compounds to be taken as external. As part of the parsing process, a preliminary classification of 'external' is applied to all compounds that consistently appear as either substrates or products in all reactions.

While many previous MPA studies, particularly on microbes, have been directly applied to genome scale networks, our approach is to first isolate a self-contained functional subnetwork to which FBA methods are then applied. This is motivated, both by:

i) the large size of the *Arabidopsis *network (2719 compounds in 2418 reactions), which would entail a combinatorial explosion of the number of EMs [25] and time complexity problems in the computation of the EMs and associated MCSs [26,27]; and

ii) the fact that only a limited subset of the secondary metabolism is relevant to flavonoid production.

For the approach to be viable, the subnetwork needs to be both self-contained (i.e. all external nodes represent plausibly buffered compounds) and complete (i.e. contain all relevant reactions from the full network).

To achieve that, we note that a subnetwork is created by cutting through some internal network nodes, in effect removing the corresponding stoichiometric constraints. That is justifiable for compounds that are known to be of sufficient supply or that are buffered by an external reservoir in the cell, such as carrier compounds like ATP and ADP. The fact that this maintains the integrity of all EMs involving such compounds, is intuitively obvious but has been more formally demonstrated using null space analysis in [28].

The following heuristic approach [29] has been designed to perform suitable partitioning of the full network, which provides the source of data and does not change in the process.

First, a list of external compounds plausibly buffered for the purposes of flavonoid metabolism is drawn up. Guidelines for this is taken from the catabolism-anabolism interface as described, for example, by Palsson [30]: fig three.two and generally recognized carrier molecules ([30]: table three.one). Additionally, the following categories of compounds are included in the list: nutrients and small environment molecules, nucleoside phosphates, amino acids, and a number of ubiquitous compounds such as chorismate, choline, UDP-glucose and malonyl-CoA. Such compounds are easily recognized in the network by the large number of reactions in which they participate.

The preliminary classification of nodes in the network mentioned above, is augmented by taking all those that correspond to compounds on this list, as external as well. The connectivity (i.e. the number of reactions in which it participates) for each node in the network is also recorded.

Next, a prototype subnetwork for flavonoid production is compiled from all reactions participating in empirically known pathways explicitly listed in the AraCyc pathways table as flavonoid related, for example, anthocyanin, flavonol, and flavanone biosynthetic pathways.

This prototype is then extended by comparing node classification and connectivity for the full and prototype networks:

1. If a node is external in the prototype but internal in the full network, its links in the full network are traced back and forwards to an acceptable external and the node becomes internal in the prototype. For example, taking the anthocyanin biosynthetic pathway as one of the starting pathways for the prototype, consider one of its starting substrates- the compound leucocyanidin. Leucocyanidin is external in the anthocyanin pathway but internal in the full AraCyc *Arabidobsis *network, where it is involved, on the one hand, in the formation of cyanidin and 2,3-trans-catechin, but itself is formed by dihydroquercetin. The related compounds and their corresponding reactions are added to the prototype and leucocyanidin becomes internal in both the revised prototype and the full network.

2. If a node is internal in both, but has larger connectivity C_f _in the full network than its connectivity C_s _in the subnetwork, then:

• If C_f _>>C_s_, the node is be reclassified as external in the prototype because there are many processes in the full network outside the subnetwork to account for buffering it. In effect, this refers to ubiquitous compounds and if we had not already recognized compounds like malonyl coA, Erythrose-4P as ubiquitous earlier in the process, they would have been classified here as external in the prototype, although they are internal, but highly connected, in the full network. A highly connected node in the full network qualifies the node to be external in the prototype.

• Otherwise, all its connections are traced back as in 1 above.

3. All reactions encountered in the traceback are added to the prototype, thus transforming a node initially not in the prototype, into an internal or an external node according to its status in the full network.

4. Classification, comparison and traceback steps 1- 3 are repeated until no reaction is added and no node is reclassified. For example, starting from our initial compound, leucocyanidin, the iterated traceback steps eventually lead to external nodes that include substrates like cinnamyl-alc or products like 2,3-trans-catechin, 2,3-cis-epicatechin, cyanidin 3-*O*-sophoroside and malonylshisonin, which are external or highly connected in the full network.

The prototype becomes a self contained subnetwork when the nodes connecting it to the rest of the full network are either external nodes in the full network, or boundary nodes that represent plausible reservoirs. Furthermore, the set of links emanating from each internal node in the subnetwork is identical in both the full network and the subnetwork. This is termed a coherent subnetwork.

**Figure 1:**
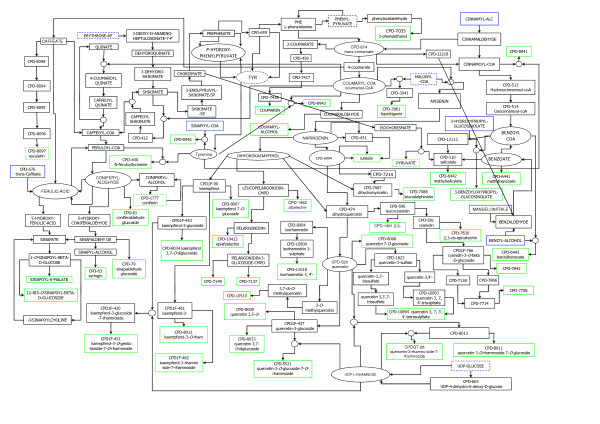
**The *Arabidopsis *flavonoid subnetwork**. In Figure 1 the compounds are drawn as rectangular and oval shapes. Black refers to internal metabolites, blue to external substrates and green to external products; dotted lines specify the metabolite taking part in other reactions outside the subnetwork. 'CPD' is an abbreviation for 'COMPOUND'. This is one form of the names (synonyms) given to compounds in the AraCyc database. For example, the name for 'liquiritigenin' in the AraCyc database and used in the data files available for downloading is CPD-3041.

There is no guarantee that steps 1 to 4 will terminate before most of the full network has been included, but if it converges to a reasonably sized subnet, this is useful for targeted analysis. In the case of the *Arabidopsis *flavonoid subnetwork, convergence was obtained for a subnet of 180 compounds and 164 reactions (refer to Figure 1). All these compounds and reactions are relevant for maintaining a coherent flavonoid subnetwork where the ABP is in full context of whole cell metabolism.

### Structural network analyses

The subnetwork is analyzed using CNA [31].

EMs [32] are calculated from the subnetwork by mathematically representing the connections between the reactions with a stoichiometric matrix and using convex analysis to identify all possible metabolic routes at steady state. The EM computations are not trivial and can be computationally challenging as shown in recent studies of time complexity [26,27] which showed that finding all EMs containing a specified reaction is hard. Each EM represents a minimal set of reactions needed to produce a certain compound. As such, the calculated EMs provide the basis for the methods used in the study and are first used to pinpoint the part of the flavonoid subnetwork that is needed to study the role of anthocyanins in the loss of colour pigmentation in plants, i.e., the ABP. The EMs responsible for the formation of anthocyanin compounds are taken to constitute the ABP and are used in the subsequent analyses.

It is important to note that reactions and compounds constitute metabolic networks so the EMs and subsequent methods used in the study are based on reactions as opposed to enzymes or genes. In order to compare with experimental observation, the reactions are related to their corresponding enzymes (interchangeable here with genes). However, these methods do not claim any knowledge of enzymes/genes or situations in which reactions occur. So, although it is necessary to discuss MCS in terms of enzymes for relating to experimental observations, the MCSs are based on the reactions themselves which are considered independently of their corresponding enzymes/genes. A consequence of this is that the same enzymes are considered separately in relation to their different reactions. This is especially important in the MCS study and will be explained further under that section and the explanations for the third table.

Three different criteria were used to prioritize enzymes targeted for suppression of colour pigmentation:

#### Reaction participation

The pleiotropic considerations such as those used by Whittal et al. can be quantified by counting the number of pathways or EMs in which each reaction and its associated ABP enzyme/gene participates. The result quantitatively shows the importance of the genes in terms of their link with other reactions and processes.

#### Minimal Cut Set (MCS)

MPA provides a more specific tool for investigating loss of function than mere participation counting: this is the concept of minimal cut sets (MCSs). The concept of MCSs was introduced in 2004 [11] and has been used to study unicellular organisms such as *Escherichia coli *[33] using their full metabolic network. This study, on the other hand, uses the MCS approach to study multicellular plant organisms using a secondary metabolism subnetwork extracted from the full network of *Arabidopsis*.

The MCSs are calculated using the concept [11] where a MCS is defined in relation to a particular network function using information from EMs. Any steady state of the network that produces a particular combination of, for example, flavonoids, can only be formed by combining the EMs associated with those flavonoid compounds. The EMs can therefore be used to compile a list of all the ways in which a particular network function can be eliminated. The network function is represented by the key reaction(s) that constitute it, designated as "objective reaction(s)"; each associated MCS consists of a minimal set of reactions elsewhere in the network that, when blocked, will remove any flux through the objective reaction(s).

Although recent studies [26] have shown that it is easy to check that a given set of reactions constitutes a cut, finding a MCS for a given set of target compounds becomes impractical in large networks. This stems from the fact that finding all EMs that use a particular reaction is NP-hard [26,27]. Moreover, from the point of view of genetic intervention it would be desirable to find a MCS of minimum size. However, [26] shows that finding cuts of minimum size without computing all EMs is also NP-hard. These considerations strongly justify the use of a reduced network for evaluating MCS, as done in our study.

The "objective reactions" for our study are those that directly form the anthocyanin compounds responsible for the function of colour pigmentation. In effect, these MCSs provide the full set of ABP reactions to block, which are related to the subsequent gene candidates for genetic changes for loss-of-function, thereby preventing anthocyanin production. Because a complex network provides many alternate pathways, there are generally several different MCSs for a single collection of objective reaction(s). All of these MCSs would be effective; however, their efficiency would differ.

Because the mathematics guarantees that the collection of MCSs is complete, we can use quantitative analysis to compare and investigate the effect that each MCS has on the remaining non-target set of EMs. Along with other different MPA methods, these effects are utilized in exploring the MCSs that would achieve loss-of-function most efficiently, and whether this was related to the position ("early" versus "late") of the genes in the ABP.

#### Fragility coefficient

Another method used in considering the best candidates for loss of colour pigmentation is the fragility coefficient calculated using the MCSs. The fragility coefficients (*fc*) provide a measure of how essential reactions are for a specific objective. The number of EMs that a gene is involved in is a good indication of its importance but it is also conceivable that a gene involved in only a small number of EMs may nevertheless be crucial for those modes. In effect, if a gene is part of a larger MCS, its *fc *will be lower as its malfunction will be less crucial for the objective reaction(s).

The *fc *of a reaction is defined as the average of the reciprocals of the number of reactions participating in an MCS [11]. For the study we want to look at the fragility coefficient of the ABP genes and how crucial they are to the pathway.

### Functional Network Analysis

#### Reaction rates/fluxes

In addition to structural considerations, the effects of losing a set of enzymes on other functions of the biochemical network are relevant in studying a loss-of-function adaptation and this is done using reaction rates. A reaction rate is the rate at which a reaction converts reactants into products; it represents the metabolic flux [33] through a reaction. The reaction rates or fluxes are used to explore the impact that each MCS has on other processes in the cell by simulating metabolic fluxes for the different MCSs and normalizing them against a reference state.

Conceptually, such impacts could be assessed by comparing the metabolic fluxes, before and after a particular MCS is imposed, by setting the fluxes through its reactions to zero through all EMs in a metabolic state that is precisely defined by measured fluxes for an individual cell. This is not practically possible because measured fluxes are not available to uniquely determine a metabolic state for the flavonoid subnetwork. Even if they were, the detailed quantitative results would strictly only apply to the single state used for the calculation.

However, a basic premise of constraints based modelling is to use partial experimental knowledge to narrow down the flux space to a compatible range, within which there is still scope for the many varied individual states produced by different environments, cell types, tissues, etc.

In the same spirit, our approach is to establish a reference state chosen to be merely representative, and plausible in the sense that flux values are not in conflict with measured values in actual cells.

Optimization (e.g. of biomass production) is often used to identify the most realistic representation of the metabolic state of a cell within the constrained region of flux space. This is not applicable for flavonoids because they are secondary metabolites not needed for basic life functions in a cell. Fortunately, however, the chosen subnetwork also supplies the ingredients for synthesizing the lignin macromolecule, which is a significant constituent of plant biomass. This allows us to relate some flux values to the well-measured overall biomass growth rate.

• Literature values for lignin monomer ratios show some variation according to plant species, tissue, developmental state and environmental stress, and similarly the lignin fraction of the biomass is variable. We make the following assumptions based on a survey of the literature [34-37]:

• The lignin composition is taken as:

370 CONIFERYL-ALCOHOL + 238 SINAPYL-ALCOHOL + 60 COUMARYL-ALCOHOL

• Taking the lignin biomass constituent as 15% [34,35] of the total biomass and the growth rate as 0.18 h^-1 ^[38], the lignin macromolecule (synthesized according to the above equation) is taken to contribute to 0.027 h^-1 ^of the primary growth of the plant.

• In the absence of measured rates for flavonoid reactions, we estimate values based on measured concentrations. We assume that the rates are proportional to the concentrations; as we only evaluate ratios between rates with and without an MCS imposed, the unknown proportionality constant cancels.

• Rates are compiled in this way for one type of plant: grapes [39,40]. Information from other plant types [41-44] are used to constrain the values of the reaction rates so they occur within a realistic range. The molar mass of the compounds is used to convert mg/kg to mmol/kg.

Table 1 contains the constraint values used for the subnetwork. These are considered preliminary values that can be modified as more information becomes available.

**Table 1:** Preliminary constraints

	Reactions	mg/kg	Reaction Rate (mmol/kg*h)
Growth rate: lignin composition [38]	mue		2.70E-02

***Flavonoid products: Grapes ***[39,40]			

***Anthocyanins***			

cyanidin 3-glucoside (cpd1f-766)	RXN1F-775	28.9	6.43E-02

cyanidin 3-p-coumaroylglucoside (cpd-7866)	RXN-8204	1.4	2.35E-03

***Flavonols***			

kaempferol-3-glucoside (cpd1f-453)	RXN1F-461	97.7	1.64E-01

quercetin-3-glucoside (cpd1f-437)	RXN1F-462	37.3	8.03E-02

***Flavan-3-ols (proanthocyanidins)***		[40.4]	[1.43E-01]

epiafzelechin: CPD-10413	RXN-9724		3.58E-02

2,3-cis-epicatechin: CPD-7630	RXN-9725		3.58E-02

afzelechin: CPD-1962	RXN-1481		3.58E-02

2,3-trans-catechin: CPD-1961	RXN-1484		3.58E-02

***Other products: Other plants ***[41-44]			**Range**

luteolin (5734-TETRAHYDROXYFLAVONE)			[0, 5.02E+01]

apigenin (cpd-431)			[0, 1.44E+01]

methylsalicylate			[0, 1.15E-02]

methylbenzoate			[0, 2.27E+01]

4-coumarate			[0, 1.84E+00]

Caffeate			[0, 3.10E-01]

Ferulate			[0, 4.50E-01]

Table 1 contains the preliminary constraint values compiled from literature [39-44], as referenced in the table, and used to generate network input fluxes that are then kept constant for the different MCSs.

Taking these constraints as well as the fixed lignin biomass production rate, CNA is allowed to calculate a feasible state of the network and this is taken as the reference state. The values of all input fluxes calculated in this state are listed in Table 2 and used subsequently for the MCS calculations as described below.

**Table 2:** Feasible input fluxes that are kept constant for the different MCSs

External input reaction	Reaction Rate
CAFFEATE-O-METHYLTRANSFERASE-RXN	8.40E+01

CONIFERIN-BETA-GLUCOSIDASE-RXN	4.51E+01

BENZYL-ALC-DEHYDROGENASE-RXN	2.98E+01

RXN-5482	1.44E+00

CINNAMYL-ALCOHOL-DEHYDROGENASE-RXN	9.26E+01

TYROSINE-DECARBOXYLASE-RXN	1.01E+02

PHEAMINOTRANS-RXN	9.26E+01

MANDELONITRILE-LYASE-RXN	2.98E+01

UDP-GLUCOSE-4,6-DEHYDRATASE-RXN	2.41E+00

DAHPSYN-RXN	9.07E+01

TYROSINE-AMINOTRANSFERASE-RXN	6.17E+01

PHENYLPYRUVATE-DECARBOXYLASE-RXN	5.00E+01

Table 2 contains the input fluxes of external substrates for a feasible state of the network. These input flux values are held constant for the subsequent simulation of the different MCSs.

Due to the varying origins of the constraints used and the arbitrary selection of a single feasible state, results based on this reference state is only expected to be indicative, not quantitatively significant. Some reassurance is obtained from the fact that the resulting flux balance system does have feasible solutions, so that the constraints are at least not mutually contradictive. A further measure to reduce reliance on details of the reference state is that only flux ratios are considered, not absolute values. It is also noted that the reference state is not meant to represent any particular physiological state, but rather a generic representation of all the capabilities of the flavonoid subnetwork. In the final analysis, the results themselves as reported in the next section gives further support to the appropriateness of this approach.

The MCS states are simulated by constraining the fluxes through the reactions constituting each MCS to zero, while maintaining the initial constraints and external inputs at the same values as for the reference state. It is conceivable that blocking the reactions in a MCS might change the fluxes exchanged with the full network, provided that such changes are compatible with flux balances in the full network. If we did allow changes to these inputs, we could not test their feasibility with just the subnet. So while we have only studied a specific case, to extend that would necessitate dealing with the complete network which does not seem practical. By keeping the influx into the network constant, the reaction rates for each MCS can be compared against the reference state. (See additional file: prelim_constr_influx for the constraints and the constant influxes)

The MCS simulated fluxes are normalized to their values in the reference state by dividing each MCS reaction rate by its rate from the reference state. This cancels out the units so we are looking at the relative impact of the MCSs on a typical (reference) state of a plant cell. A similar analysis was done without the constraints, to check that the relative impact is not just specific to the chosen reference state; it was found that the flux ratios were generally the same whether the preliminary constraints were used or not.

To represent the results the external metabolite products are grouped into seven flavonoid groups and four non-flavonoid groups. The flavanoid groups are flavanols, flavanones, flavones, proanthocyanidins, leucoanthocaynidins, anthocyanins and flavonols which is further split into two groups: flavonols1 consisting of kaempferol derivatives and flavonols2 consisting of quercetin derivatives. The average relative fluxes through the end reactions of the EMs producing these external products enable us to study the impact that the MCSs have on other processes.

## Results and Discussions

### The subnetwork

The extracted flavonoid subnetwork shown in Figure 1 is quite complex, containing all cell compounds and reactions related to flavonoid metabolism and necessary to sustain it as an independent network.

The flavonoid subnetwork has more metabolites than reactions; it consists of 180 metabolites and 164 reactions. This illustrates a common feature of plant secondary metabolites where "decorating" (glycosylating, acylating, and methylating) enzymes are of low substrate specificity, enabling a single reaction to utilize multiple substrates to produce different products [12,17].

In addition, the 359 EMs calculated from the flavonoid network is quite low compared to previous studies that concentrated on the primary metabolism of microbes [8,9]. These had more reactions than metabolites and, consequentially, very high numbers of EMs, for example the 2.4 million EMs calculated from 112 reactions and 89 compounds in Escherichia coli [32]. The low number of EMs in the flavonoid subnetwork indicates that the network is highly constrained, which makes sense considering that the number of reactions determines the dimension of the EM subspace, while the number of internal compounds represents the number of constraints. One biological interpretation of this observation is that the network mainly comprises secondary metabolites which have more specific functions. Consequently, the metabolites in the flavonoid network are involved in fewer reactions, compared to primary compounds fundamental to plant growth and thus involved in many reactions. These speculations could be further investigated by looking at other extracted pathways representing different types of metabolism and comparing the number of reactions and EMs to see if they support the theory. This provides possibilities for future studies.

From the 359 EMs calculated from the subnetwork, 203 lead to non-flavonoid products, whilst 156 lead to flavonoid compounds of which there are seven subclasses. These are: i) anthocyanins; (ii) flavanols; iii) flavanones; iv) flavones; v) flavonols; vi) leucoanthocyanidin and vii) proanthocyanidins. Details of these flavonoids can be seen in [45-47]. Four non-flavonoid groups are identified: i) lignin [35]; ii) benzenoids [48,49]; iii) amides; and iv) coumarins [50,51]. The rest of the non-flavonoid compound products are classified as 'others': compounds that form ambiguous products, either because the products are also substrates in the synthesis of other compounds (e.g., chorismate [52] and tyrosine [53,54]), or the EMs form futile cycles.

**Figure 2:**
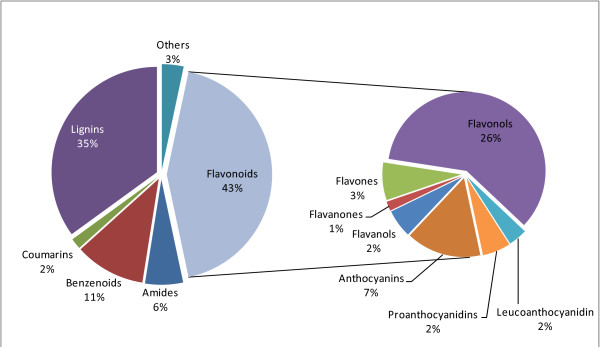
**Classification of EMs calculated from the flavonoid subnetwork**. Figure 2 classifies the EMs into different flavonoid and non-flavonoid groups according to the phenotypic functions of the products in the network.

Figure 2 classifies the EMs into different flavonoid and non-flavonoid groups according to the phenotypic functions of the products in the network as described above.

The focus of this study is on the anthocyanin compounds and their role in plant colour pigmentation. As shown in Figure 2, 7% of the total number of EMs in the subnetwork lead to the formation of anthocyanins and their derivatives. This amounts to 24 EMs taken to constitute the anthocyanin biosynthetic pathway (ABP).

### The Anthocyanin Biosynthetic Pathway (ABP)

**Figure 3:**
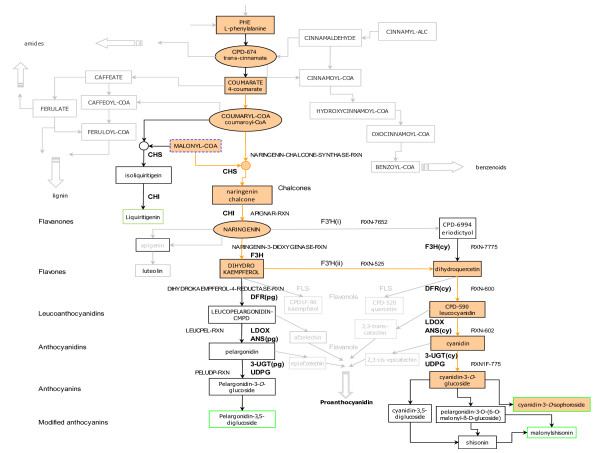
**The ABP nodes extracted from the reconstructed flavonoid subnetwork of *Arabidopsis***. Figure 3 is a detailed part of the self-contained subnetwork showing the subset of nodes or metabolites in the 24 EMs that constitute the ABP. The compound groups that are not directly related to anthocyanins, are shown as greyed-out side branches from the ABP. Highlighted is an example of one anthocyanin EM leading to the formation of cyanidin 3-*O*-sophoroside. The names of compounds and reactions are as assigned in the AraCyc database [63]. *Enzymes*: CHS: Chalcone Synthase; CHI: Chalcone isomerase; F3H: Flavanone 3-hydroxylase; F3'H: Flavanone 3'-hydroxylase; DFR: Dihydroflavonol reductase; ANS: Anthocyanidin synthase; LDOX: Leucoanthocyanidin dioxygenase; FLS: Flavonol synthase; UDPG: UDP-glycosyltransferase; 3-UGT: Anthocyanidin 3-*O*-glucosyltransferase; 5-GT: Anthocyanin 5-*O*-glucosyltransferase (Hexosyltransferase); Ss5MaT1: Anthocyanin 5-*O*-glucoside 6-*O*-malonyltransferase (Acyltransferase); 2.4.1.- Hexosyltransferases; 2.3.1.- Acyltransferases: Transferring groups other than aminoacyl groups. (Pg) indicates enzymes corresponding to reactions related to pelargonidin-type compounds; (Cy) indicates enzymes corresponding to reactions related to cyanidin-type compounds.

A detailed part of the subnetwork showing the subset of nodes or metabolites involved in the 24 EMs relevant for the ABP is shown in Figure 3. This is not a coherent self-contained subnetwork, as discussed earlier for Figure 1, but merely an extract for display purposes. The compound groups that are not directly related to anthocyanins, are shown as greyed-out side branches from the ABP.

As shown in Figure 3, *Arabidopsis *contains two anthocyanin compounds responsible for colour pigmentation: pelargonidin-3*-O-*glucoside (Pg) and cyanidin-3*-O-*glucoside (Cy). The number of EMs (routes) is higher for Cy-type anthocyanin than Pg-type anthocyanin because EM calculation depends on the structure of the metabolic network and how the compounds are linked by reactions. As shown in Figure 3, two earlier metabolic reactions (RXN-7652 & RXN-525) both lead to Cy-type anthocyanins compared to one reaction (NARINGENIN-3-DIOXYGENASE-RXN) leading to Pg-type anthocyanins. In addition, there are more modifying reactions involved post-Cy anthocyanin than there are post-Pg anthocyanin. These differences result in more EMs (routes) for Cy-type anthocyanin because: i) EMs can go through the reaction RXN-7652 or RXN-525, whereas to form Pg-type anthocyanin you can only go through the NARINGENIN-3-DIOXYGENASE-RXN reaction and ii) there are more modifying reactions post cyanidin-3-*O-*glucoside than there are post pelargonidin-3-*O*-glucoside.

Further modification of the anthocyanin compounds (by glycosylation, acylation and/or methylation) is illustrated in Figure 3 and in the number of EMs but was not taken into account for the MCS study as these late-stage modified compounds are concerned with the fine adjustment of colors [55,56]. We, on the other hand, only want to investigate the loss of plant colour pigmentation in relation to certain biological studies [5,6,57,58]. Colour pigmentation occurs when an anthocyanin compound, Pg and/or Cy is formed so, in terms of eliminating pigmentation, the MCSs can involve any reaction before the anthocyanins are formed. Also, even though some Pg and Cy-related reactions may share the same enzyme/gene we don't know enough about how and when they catalize the corresponding reactions so each reaction is treated independently, even if they correspond to the same enzyme(s). In view of these it is necessary to distinguish between Pg and Cy-type anthocyanins for MCSs consisting of genes further down the pathway where the formation of the separate pigments need to be blocked to eliminate pigmentation.

For the metabolic reactions in the ABP, the main related genes/enzymes to be studied are: CHS, CHI, F3H, F3'H, DFR, LDOX/ANS [59,60], and UDPG/3-UGT [61] (refer to Figure 3 caption for details of genes).

Genes such as FLS [60] are not directly related to anthocyanins but share the pathway and lead to the production of other flavonoid compounds such as flavonols (quercetin and kaempferol) which also play a role in co-pigmentation [62]. The genes, 5-GT, Ss5MaT1, etc., are involved in later reactions for anthocyanin modifications [55,56]. Since all these genes provide colour change or modification to the anthocyanin compounds, they take effect after the formation of anthocyanin compounds and do not play a role in anthocyanin production, so are not included in the group of genes looked at for eliminating colour pigmentation (anthocyanin production).

### Structural Network Analysis

#### Gene participation (connectivity)

**Figure 4:**
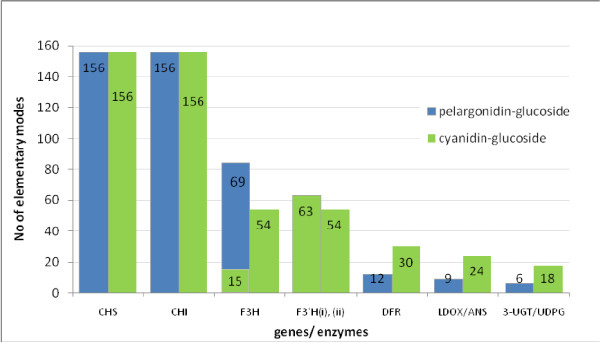
**Participation of ABP genes**. Figure 4 illustrates the number of EMs the two *Arabidopsis *anthocyanin compounds participate in. The genes on the graph correspond to their positions in the ABP sequence: the earliest gene in the ABP sequence (CHS) starts on the left hand side and the latest genes (UDPG/3-UGT) are on the right hand side. Refer to Figure 3 for details and explanation of abbreviated gene names.

The first network property looked at is the connectivity of the anthocyanin genes as shown in Figure 4. The graph shows the importance of the anthocyanin genes in terms of their connectivity to other reactions and processes. The graph starts with the "early" genes on the left hand side and finishes with the "late" genes on the right hand side, in relation to their position in the ABP sequence.

The results in Figure 4 agree with those of Whittal et al [5] regarding the pleiotropic function of the "early" genes and its decrease as you move down the ABP; it provides a more quantitative representation of their observations by actually determining the number of EMs to which the genes are connected. The "earliest" genes CHS and CHI are related to 156 EMs while the "latest" genes together only occur in the 24 EMs responsible for anthocyanin formation. The information provides a quantitative support for the explanation of "late" genes being targeted for loss-of-function mutations. However, it is not enough to say that gene connectivity is a deciding factor in determining which genes to target for loss-of-function because being pleiotropic does not mean for certain that the gene will not be targeted for loss-of-function, and vice versa. Moreover, the absence of a single enzyme may not be enough to prevent pigmentation for certain EMs responsible for anthocyanin formation. MCS, on the other hand, is directly focused on a specific objective function, so by specifying the objective as loss-of-function, it can be used to determine target gene sets that would guarantee loss-of-function.

#### Candidates for loss of colour pigmentation

Table 3 shows the set of MCSs or ABP gene candidates for genetic changes needed to eliminate the formation of the initial anthocyanin compounds. In order to ensure a full list of MCSs for pigmentation loss, it is necessary to distinguish between the two types of anthocyanins (Pg and Cy-types). This is for cases where MCSs consist of genes further down the pathway where it is still possible to eliminate pigmentation.

**Table 3:** ABP minimal cut sets (MCSs) with corresponding ABP gene(s) and reactions.

MCS	N-3-D-RXN	LEUCPEL-RXN	D-4-R-RXN	RXN-525	APIGNAR-RXN	RXN-600	RXN-602	RXN-7652	PELUDP-RXN	RXN1F-775	RXN-7775	N-C-S-RXN	Total rxns	Unaffected	Genes/enz in MCS
**1**	0	0	0	0	1	0	0	0	0	0	0	0	1	3	CHI

**2**	0	0	0	0	0	0	0	0	0	0	0	1	1	3	CHS

**3**	1	0	0	0	0	1	0	0	0	0	0	0	2	57	F3H, DFR(cy)

**4**	1	0	0	0	0	0	0	1	0	0	0	0	2	9	F3H, F3'H(i)

**5**	0	1	0	0	0	1	0	0	0	0	0	0	2	126	ANS(pg), DFR(cy)

**6**	0	0	1	0	0	1	0	0	0	0	0	0	2	123	DFR(pg), DFR(cy)

**7**	1	0	0	0	0	0	1	0	0	0	0	0	2	60	F3H, ANS(cy)

**8**	0	1	0	0	0	0	1	0	0	0	0	0	2	132	ANS(pg), ANS(cy)

**9**	0	0	1	0	0	0	1	0	0	0	0	0	2	129	DFR(pg), ANS(cy)

**10**	0	0	0	0	0	1	0	0	1	0	0	0	2	129	DFR(cy), 3-UGT(pg)

**11**	0	0	0	0	0	0	1	0	1	0	0	0	2	135	ANS(cy), 3-UGT(pg)

**12**	1	0	0	0	0	0	0	0	0	1	0	0	2	63	F3H, 3-UGT(cy)

**13**	0	1	0	0	0	0	0	0	0	1	0	0	2	138	ANS(pg), 3-UGT(cy)

**14**	0	0	1	0	0	0	0	0	0	1	0	0	2	135	DFR(pg), 3-UGT(cy)

**15**	0	0	0	0	0	0	0	0	1	1	0	0	2	141	3-UGT(pg), 3-UGT(cy)

**16**	1	0	0	0	0	0	0	0	0	0	1	0	2	18	F3H, F3H(cy)

**17**	0	1	0	1	0	0	0	1	0	0	0	0	3	39	ANS, F3'H, F3'H

**18**	0	0	1	1	0	0	0	1	0	0	0	0	3	36	DFR, F3'H, F3'H

**19**	0	0	0	1	0	0	0	1	1	0	0	0	3	42	F3'H, F3'H, 3-UGT(pg)

**20**	0	1	0	1	0	0	0	0	0	0	1	0	3	48	ANS, F3'H, F3H(cy)

**21**	0	0	1	1	0	0	0	0	0	0	1	0	3	45	DFR, F3'H, F3H(cy)

**22**	0	0	0	1	0	0	0	0	1	0	1	0	3	51	F3'H(ii), 3-UGT(pg), F3H(cy)

***fc***	0.50	0.43	0.43	0.33	1	0.50	0.50	0.38	0.43	0.50	0.38	1			

**Enzymes**	1	2	3	4	5	6	7	8	9	10	11	12			

Table 3 shows the set of MCSs or ABP gene candidates for genetic changes needed to eliminate anthocyanin production. Some reactions in the top row have been abbreviated and the numbers in the bottom row represent enzymes corresponding to the reactions in the top row; the details are as follows:

Reactions (top row):

N-3-D-RXN: NARINGENIN-3-DIOXYGENASE-RXN

D-4-R-RXN: DIHYDROKAEMPFEROL-4-REDUCTASE-RXN

N-C-S-RXN: NARINGENIN-CHALCONE-SYNTHASE-RXN

Unaffected: Non-affected flav EMs

Enzymes (bottom row):

1: F3H; 2: ANS/LDOX (pg); 3: DFR(pg); 4: F3'H(ii); 5: CHI; 6: DFR(cy); 7: ANS/LDOX (cy); 8: F3'H(i); 9: 3-UGT/UDPG (pg); 10: 3-UGT/UDPG (cy); 11: F3H(cy); 12: CHS

The reactions directly forming the anthocyanin compounds (PELUDP-RXN and RXN1F-775) are identified as the objective functions for which the MCSs are determined. In the last column, parallel enzymes are listed by a single representative: ANS & LDOX corresponding to LEUCPEL-RXN as ANS(pg), ANS & LDOX corresponding to RXN-602 as ANS(cy); 3-UGT & UDPG corresponding to PELUDP-RXN as 3-UGT(pg) and 3UGT & UDPG corresponding to RXN1F-775 as 3-UGT(cy). *fc *- fragility coefficient. (Refer to Figure 3 legend or *List of abbreviations used*, for explanation of the abbreviated enzymes.)

The reactions directly forming the anthocyanin compounds (PELUDP-RXN and RXN1F-775) are identified as the objective functions for which the MCSs are determined.

**Figure 5:**
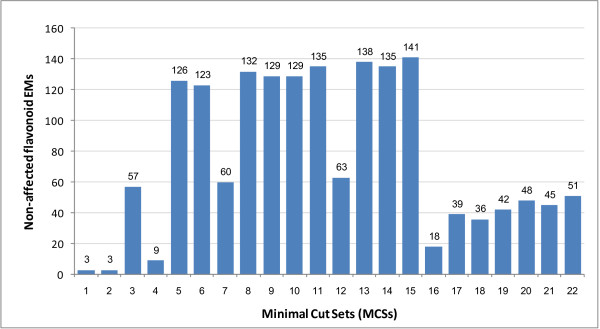
**The number of EMs not affected by each MCS**. The number of EMs (not including the 24 anthocyanin EMs) not affected when the reaction(s) that constitute each MCS is/are blocked. The MCS numbers on the x-axis correspond to the MCS numbers in the first column of Table 3.

The 22 MCSs shown in Table 3 identify the different combinations of ABP genes to block in order to eliminate colour pigmentation. The last column shows the ABP gene(s) involved in each MCS with the second last column showing the number of EMs (141 non-anthocyanin flavonoid EMs) not affected by each of these cut sets. This is shown graphically in Figure 5 which provides a means of prioritizing MCS candidates by their effect on other processes. Figure 5 shows a graph of the impact that each MCS has on the remaining flavonoid EMs. The MCSs are based on reactions so the same gene(s) corresponding to different reactions are treated independently and assumed to act independently for their own corresponding reactions.

At the top of the list is MCS15 which does not affect any other mode except the 24 anthocyanin EMs. As shown in Figure 3 and Table 3, this MCS consists of the 3-UGT(pg) and 3-UGT(cy) gene associated with both objective functions/reactions so this would be the best gene to block to eliminate anthocyanin production without affecting other EMs or the production of other compounds. The second best cut set is MCS 13 (genes ANS(pg) & 3-UGT(cy)) with 138 EMs not affected. One of the genes (3-UGT(cy)) corresponds to an objective function.

The MCS result illustrated in Figure 5 could be used together with the previous results represented in Figure 4 which shows the connectivity of these genes in terms of the number of EMs that they participate in along with their sequential positions in the ABP. The gene participation graph shows the pleiotropic function of the earliest genes in the ABP, CHS and CHI, which are involved in all flavonoid EMs. As you move down the pathway sequence, the number of EMs in which the genes are involved, decreases; the latest gene (3-UGT(pg) & 3-UGT(cy)) is only involved in the anthocyanin EMs. Figure 4 also shows that, next to MCS15, the two genes in MCS13, ANS (pg) and 3-UGT (cy), would cause minimum disturbance because they are only involved in 27 EMs- the 9 Pg anthocyanin modes plus an additional 18 for Cy.

On the other hand, it could be more efficient to block an 'early' gene, which argues for MCSs 11 (ANS(cy) & 3-UGT(pg)) and 14 (DFR(pg) & 3-UGT(cy)) with 135 non-affected EMs. While these eliminate 3 additional modes, they may compensate for it by moving the cut higher up the pathway, although these MCSs contain at least one objective function gene. The next five MCSs that do not contain any objective function genes but still make good candidates are: MCS8 (ANS(pg) & ANS(cy)) with 132 non-affected EMs, MCS9 (DFR(pg) & ANS(cy)) and MCS10 (DFR(cy) & 3-UGT(pg)) with 129 non-affected EMs, MCS5 (ANS(pg) & DFR(cy)) with 126 non-affected EMs, and MCS6 (DFR(pg) & DFR(cy)) with 123 non-affected EMs. The number of non-affected EMs then drops to the 60 s or lower.

The above analysis gives independent insight into the reasons for the observed molecular convergence in the evolution of colour pigmentation losses by providing a complete listing of all options open to the plant for suppressing anthocyanin production, as well as characterization of the impact each mutation has on the production of other compounds. It shows a more quantitative and therefore more differentiated characterization of the enzyme roles through the exhaustive nature of the mathematical analysis of network structure. Fragility coefficients (*fc*) could provide further information on target genes by looking at how essential they are to the pathway.

#### How essential are the ABP genes?

**Figure 6:**
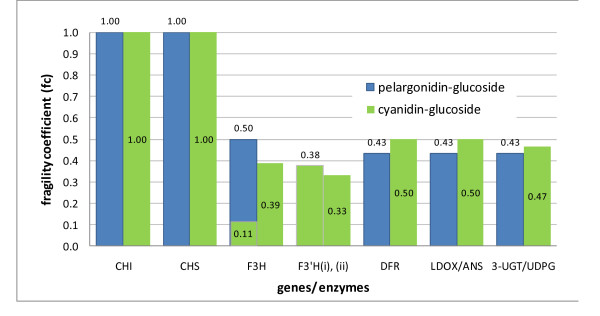
**Fragility coefficients (*fc*) of the ABP genes**. The fragility coefficients of the ABP genes in the production of each of the anthocyanidin glucosides. Refer to the 2^nd ^bottom row of Table 3 for details of *fc *values.

The two bottom rows of Table 3 respectively show the *fc *and the genes corresponding to the reactions in the first row (also shown in Figure 3). A graphical representation of the ABP genes' *fc *is shown in Figure 6.

When compared with Figure 4, Figure 6 also shows CHS and CHI as crucial to the ABP but, unlike gene participation, shows later genes, DFR and ANS/LDOX along with UDPG/3-UGT, as more essential for anthocyanin production than the earlier F3H and F3'H genes. It is to be expected that 3-UGT/UDPG are more essential as they directly form the anthocyanin compounds but DFR and ANS/LDOX are more essential than the earlier F3H and F3'H genes because they are involved in MCSs with fewer reactions, so the network structure identifies them as more crucial for anthocyanin formation.

Considering all the issues highlighted in the analyses so far, the genes ANS/LDOX and DFR corresponding to MCSs 5 and 9 could be considered as having favourable characteristics: they participate in a low number of EMs so don't affect the production of too many compounds; they occur earlier in the pathway and; they are more essential for anthocyanin production (higher *fc*) than other earlier genes. This supports the observation by Whittall et al [5] of the genes ANS and DFR being targeted and co-regulated for eliminating floral pigmentation in *Aquilegia*.

The genes making up the MCSs need to be suppressed together to be effective so the concept of MCS goes well with that of a regulatory gene regulating the expression of a set of structural genes. Several regulatory genes are known to regulate the expression of sets of structural genes in the ABP e.g., DFR and all later genes are co-regulated in Petunia and ANS onwards in antirrhinum [6]. These sets are larger than the 2-member sets determined in this study. That may be because the observations in [6] relate to colour changes rather than complete loss and to study it in detail would need the use of different objective functions; this is left for future study.

### Functional Network Analysis

#### Implications of eliminating the ABP genes

In the absence of detailed data on metabolic fluxes in a particular tissue of *Arabidopsis *under particular conditions, a reference state is constructed in which as many fluxes as possible are constrained to values that are plausible in the sense of having been measured for other species or tissues. While this reference state is somewhat artificial, it is important because the assumptions that constitute it are based on real experimental facts and therefore provide an initial benchmark for the credibility of the subnetwork or the model.

The reference state addresses all situations represented by the subnetwork. For example, it takes the presence of lignin-related compounds as an indication of flavonoid metabolism being closely linked with plant growth and as such uses them to constrain the subnetwork to account for plant biomass and primary growth. Other real-life based constraints on the metabolism of flavonoid compounds together form validation of the subnetwork model which is further justified by the fact that it gives a feasible solution, in other words it contains no contradictions in terms of flux balances.

The initial assumptions that constitute the reference state do constrain the subnetwork and it may be that certain assumptions may not apply in certain environmental situations or in certain plant cells so the reference state should be seen as representing what the flavonoid subnetwork is realistically capable of, rather than any specific state. As such, it allows exploration of the effects of a given MCS on all functions of the subnet, not just those that are active in particular conditions.

In the second step of the simulation, the inputs to the subnet are kept fixed at the values required for the reference state. The rationale behind this is that those inputs are supplied by the rest of the metabolic network and, in effect, it is assumed that the rest of the metabolism is not affected by readjustments inside the flavonoid subnetwork. In the absence of information about such effects, this assumption is at least a reasonable starting point when comparing plants growing under the same external conditions but with or without colour pigmentation.

**Figure 7:**
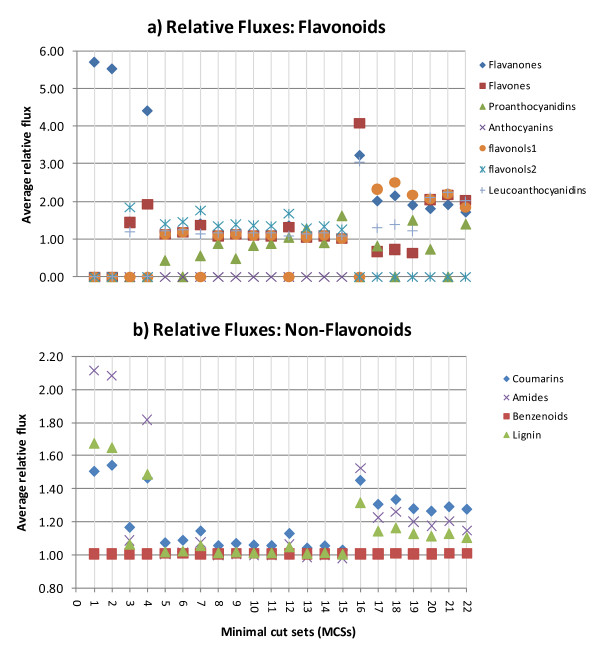
**Relative Fluxes (reaction rates)**: **a) - Average reaction rates producing the flavonoid product compound classes**. Average relative fluxes (reaction rates) producing the external flavonoid compound classes for each MCS. Flavonols1: kaempferol derivatives; Flavonols2: quercetin derivatives. **b) - Average reaction rates producing the non-flavonoid compound groups**. Average relative fluxes (reactions rates) producing the non-flavonoid compound groups for each MCS.

Figures 7(a & b) show the relative fluxes simulated for different MCSs and demonstrate the effect that eliminating colour pigmentation, by suppressing certain ABP genes, has on other reaction rates.

The reaction rates shown in Figures 7a) and Figure 7b) provide extra information about MCSs:

• MCS 1 and 2 eliminate anthocyanin production by respectively constraining the CHI and the CHS reactions to zero (refer to Table 3). As shown by the relative fluxes in Figure 7a), this also effectively eliminates all other flavonoids (fluxes = 0) except for the flavanone liquiritigenin and the non-flavonoid compound products. Benzenoids are hardly affected;

• MCS 4 and 16 have, to a lesser extent, similar effects as MCSs 1 and 2 but with an additional increase in flavone fluxes especially by MCS 16 which also shows a corresponding flux increase in leucoanthocyanidins. All other flavonoids are eliminated;

• MCSs 13 & 15 show an increased reaction flux for PAs and flavonols and would make likely candidates for stages in plants such as early berry development [57], where PA and flavonol promoters are activated but not the anthocyanins.

• MCSs 17 to 22 are good candidates to consider for when you are looking at ways to eliminate pigmentation that will also eliminate quercetin related flavonols (and PAs as well for MCSs 18 & 21) but increase fluxes of kaempferol related flavonols.

In general, the reaction rates show that non-flavonoid compounds are not much affected by the elimination of anthocyanins. Even for MCSs 1 and 2 which require the elimination of the early ABP genes that result in the elimination of most of the flavonoids, benzenoid production is not affected. This provides a quantified explanation of experimental observations such as that by Spitzer et al [58] where they showed that the suppression of the anthocyanin pathway, via CHS silencing, dramatically reduced flavonoids but did not affect scent (benzenoids) production.

The above FBA provides a quantified measure of the impact that the different MCSs would have on the production and processes of other flavonoids and related compounds. They provide a more informative way of considering favourable target genes for eliminating colour pigmentation, and show that MCS options that appear similar in terms of structural measures, can have opposite quantitative impacts. For example, looking at genes ANS & DFR (MCSs 5 & 9) that were considered favourable target genes for eliminating colour pigmentation from the previous network structural analysis, the FBA shows that the effect of their MCSs on reaction fluxes is mainly to decrease PA. MCSs 13 & 15, on the other hand, lead to an increase in fluxes through the reactions mainly producing PAs and flavonols. Both effects have been observed in different systems. This additional information suggests that different MCSs suit different cell types or environmental conditions and could be utilized to identify which MCS would work best to achieve a certain outcome.

While the reference state used for the reaction rate analysis is admittedly only one out of a possible range of feasible states, the general principle demonstrated by the results is that various MCSs that are by construction equivalent regarding a set of target products, can have quite different repercussions on the flux distribution in the rest of the network.

## Conclusions

In the main focus of the study, three methods (gene participation, MCSs, and reaction rates) are employed to consider target ABP reactions and their corresponding genes for eliminating colour pigmentation. On their own, gene participation and reaction rates cannot be used as a basis for identifying the genes to target but they do provide an idea of the importance of, and interplay between, genes from a whole cell perspective. This information, along with MCSs, proves very useful in considering target genes for blocking colour pigmentation.

The MCS method is the most important because it offers a list of the set of loss-of-function target genes that would eliminate anthocyanin production. By using the other methods to investigate these MCSs, we are able to analyze the properties of the corresponding genes and the impact that their suppression would have on other processes in the network. The fact that the impacts we demonstrate do coincide with actually observed distinct patterns of flavonoid production supports the simplifying assumption about our reference state and the analysis using reaction rates.

The first two methods analyze the structure of the network to determine EMs and make predictions on the capability of the genes in terms of the network structure. When considering the MCS results and initially looking at the impact that each MCS had on other EMs, MCS 15 stands out as containing the best set of genes to target. However, looking at the different MCSs in relation to gene participation and fragility coefficients, the case arises that it might not be so good in certain circumstances and the argument develops for the possibility of other MCSs making better candidates for different situations related to loss of colour pigmentation in plants.

The third method takes the analysis a step further by looking at the functionality of the genes in terms of simulated reaction rates for different MCSs. By using all the three methods together, we are able to consider different possibilities and relate these to experimental observations that have been made. For example, MCSs 5 and 9 that respectively require the simultaneous suppression of genes DFR(cy)/ANS(pg) and DFR(pg)/ANS(cy) to eliminate colour pigmentation, correspond to observational results of the co-regulation of the DFR and ANS genes for eliminating floral pigmentation in *Aquilegia *[5]; MCS 2 corresponds to experimental findings [58] that showed that the suppression of the early gene CHS eliminated nearly all flavonoids but did not affect the production of volatile benzenoids responsible for floral scent; MCSs 13 and 15 correspond with experimental findings [57] in which, during early grape berry development, PA and flavonol promoters were activated but not the anthocyanins.

The results of this study provide a comprehensive list of all the different MCSs possible, according to metabolic network information available to date. It is obvious that these different MCSs would be best for different plants, different cells, and different conditions and may correspond to regulatory genes. From the results we are also able to understand more about the interplay between the MCSs and other metabolic processes in the cell; knowledge which enables us to relate which MCS would correspond best with which plant, cell or condition. Being able to relate the different MCSs to experimental observations, not only provides credence to the mathematical approach, but could also assist with the design of experiments that would more quickly achieve the required results. It also gives credibility to use MCSs to analyse and predict the metabolic functionality of less understood pathways.

## List of Abbreviations Used

3-UGT: Anthocyanidin 3-O-glucosyltransferase; ABP: Anthocyanin Biosynthetic Pathway; ANS: Anthocyanidin synthase; C_f_: Connectivity in the full network; C_s_: Connectivity in the subnetwork; CHI: Chalcone isomerise; CHS: Chalcone synthase; CNA: CellNetAnalyzer; Cy, cy: cyanidin-3-O-glucoside; DFR: Dihydroflavonol reductase; EM: Elementary Mode; F3H: Flavanone 3-hydroxylase; F3'H: Flavanone 3'-hydroxylase; FBA: Flux Balance Analysis; fc: fragility coefficient; FLS: Flavonol synthase; LDOX: Leucoanthocyanidin dioxygenase; MCS: Minimal Cut Set; MPA: Metabolic Pathway Analysis; Pg, pg: pelargonidin-3-O-glucoside; UDPG: UDP-glycosyltransferase.

## Competing interests

The authors declare that they have no competing interests.

## Authors' contributions

STC participated in the design of the study, carried out the analysis and drafted the manuscript. WSV helped in the design of the study, the analysis, and the draft of the manuscript. All authors read and approved the final manuscript.

## Supplementary Material

Additional file 1 **AC8_subnet.sbml**. This is an sbml [23] file representing the AraCyc flavonoid subnetwork model. Click here for file

Additional file 2 **metabolites**. A file containing the metabolites and their attributes, downloaded from the AraCyc database [19]. Each row defines a metabolite with its parameter, i.e., <identifier> <full name>. Click here for file

Additional file 3 **prelim_constr_influx.val**. A file specifying the preliminary constraints. This can be uploaded and displayed in the CellNetAnalyzer flavonoid network project using the 'Scenario' feature under the 'CellNetAnalyzer' menu. Click here for file

Additional file 4 **reactions**. A file containing details of the reactions in the flavonoid subnetwork, downloaded from AraCyc. Each row in the file defines a reaction and its properties: <reaction identifier> <reaction equation> | <default value: # if unknown> <Par1: rate minimum> <Par2: rate maximum> <Par3: objective function coefficient 1- exclude, 0- include> <x-Pos: pixel coordinates> <y-Pos: pixel coordinates> <map number: network map where the box appears> <text box type: 1- editable, 2- noneditable >. Click here for file
